# Efficacy of Topical Corticosteroid Therapy in Chronic Rhinosinusitis Post-endoscopic Sinus Surgery: A Narrative Synthesis of Randomized Controlled Trials

**DOI:** 10.7759/cureus.92274

**Published:** 2025-09-14

**Authors:** Shahad F Alharbi, Elaf M Al-Juaid, Saad A Alghamdi, Dareen A Abdullah, Abdulaziz a Al Hatem, Abeer s Almalki, Raghad n Alasiri, Shaima s Alrabie, Omima t Wafa, Lama b Almutairi, Amal m Bamehriz, Rahaf k Badawi, Raed Almutairi, Faris Alnosayan

**Affiliations:** 1 College of Medicine and Medical Sciences, Qassim University, Unaizah, SAU; 2 College of Medicine and Surgery, Taif University, Taif, SAU; 3 College of Medicine, Taibah University, Medina, SAU; 4 Faculty of Medicine, University of Jeddah, Jeddah, SAU; 5 College of Medicine, King Khalid University, Abha, SAU; 6 College of Medicine, King Saud Bin Abdulaziz University for Health Sciences, Jeddah, SAU; 7 College of Medicine, King Abdulaziz University, Jeddah, SAU; 8 Department of Otorhinolaryngology, King Fahad Specialist Hospital, Buraidah, SAU

**Keywords:** chronic rhinosinusitis, drug-eluting stents, endoscopic sinus surgery, inflammation, postoperative healing, topical corticosteroids

## Abstract

Chronic rhinosinusitis (CRS) is a frequent inflammatory disorder that can markedly impair patients’ quality of life. Endoscopic sinus surgery (ESS) remains the treatment of choice for refractory cases, but postoperative inflammation and adhesion formation are still major concerns. Corticosteroids are often prescribed to address these complications, although systemic administration carries the risk of adverse effects. This has led to growing interest in localized, topical corticosteroid therapies. This review followed the Preferred Reporting Items for Systematic Reviews and Meta-Analyses (PRISMA) framework and was prospectively registered in the International Prospective Register of Systematic Reviews (PROSPERO). Eligible studies included randomized controlled trials (RCTs) comparing topical corticosteroid interventions with standard postoperative care or placebo in CRS patients undergoing ESS. Searches were conducted in PubMed and Google Scholar up to July 2024. Data extraction and quality appraisal were independently performed by two reviewers, with disagreements resolved by a third reviewer. Of the 141 screened records, six RCTs comprising 530 participants met the inclusion criteria. Pooled analysis indicated that topical corticosteroids enhanced mucosal recovery, decreased adhesion (synechiae) rates, and reduced polyp recurrence. Drug-eluting stents and budesonide irrigation demonstrated the most notable benefits without significant systemic complications. Patient-reported improvements were consistently documented using the Sino-Nasal Outcome Test-22 (SNOT-22) and Lund-Kennedy scoring systems. Topical corticosteroid use following ESS offers clear benefits by promoting healing and minimizing complications, with limited systemic risks. Nevertheless, heterogeneity across trials highlights the need for further well-designed studies with standardized methodologies to strengthen the evidence base and guide routine clinical use.

## Introduction and background

Chronic rhinosinusitis (CRS) is a long-standing inflammation of the nasal and paranasal sinus mucosa lasting more than 12 weeks. It represents a major health concern worldwide, with significant socioeconomic consequences [1,2]. In North America and Europe, CRS affects an estimated 5-12% of the population, highlighting its clinical and economic burden [1,3]. The disease impacts individuals across all age groups and often necessitates a combination of medical management and surgical intervention. Endoscopic sinus surgery (ESS) is a widely accepted, minimally invasive technique for patients who do not respond adequately to medical therapy [4,5]. Functional ESS (FESS) is primarily performed to enhance the effectiveness of subsequent medical therapy, particularly topical corticosteroids, by improving sinus ventilation and facilitating direct drug delivery to the mucosa.

Postoperative management after ESS has traditionally included systemic and topical corticosteroids. Corticosteroids are known to accelerate mucosal healing, reduce edema, and limit bacterial growth, which together improve surgical outcomes [6,7]. However, systemic administration is associated with considerable adverse effects, including hyperglycemia, osteoporosis, mood disturbances, avascular necrosis, and cataract development [6].

As an alternative, localized corticosteroid delivery - either through topical application or intraoperative drug-eluting devices - has been investigated. These approaches allow for controlled release at the surgical site, supporting mucosal recovery, preserving sinus patency, and reducing adhesion formation, with minimal systemic exposure [8-10].

The purpose of this narrative review is to summarize and critically discuss the findings of randomized controlled trials (RCTs) that have evaluated the role of topical corticosteroids in enhancing healing and reducing postoperative complications in CRS patients undergoing ESS.

## Review

Methods and materials

Review of the Literature

For this narrative review, we structured the process according to principles inspired by Preferred Reporting Items for Systematic Reviews and Meta-Analyses (PRISMA) to minimize selection bias [11]. The protocol was prospectively recorded in the International Prospective Register of Systematic Reviews (PROSPERO) under the ID: CRD42024569582 [12]. Ethical approval was not required for this type of study. In July 2024, a comprehensive literature search was carried out using PubMed and Google Scholar databases. The following search terms and combinations were applied: ((“chronic rhinosinusitis” OR CRS) AND (“endoscopic sinus surgery” OR ESS) AND (“topical corticosteroid*” OR “steroid delivery” OR “steroid eluting” OR “drug eluting”)).

Eligibility of articles was evaluated using the PICOTS (population, intervention, comparison, outcome, timing, setting) framework.

Methodology for Selecting Studies

**Figure 1:**
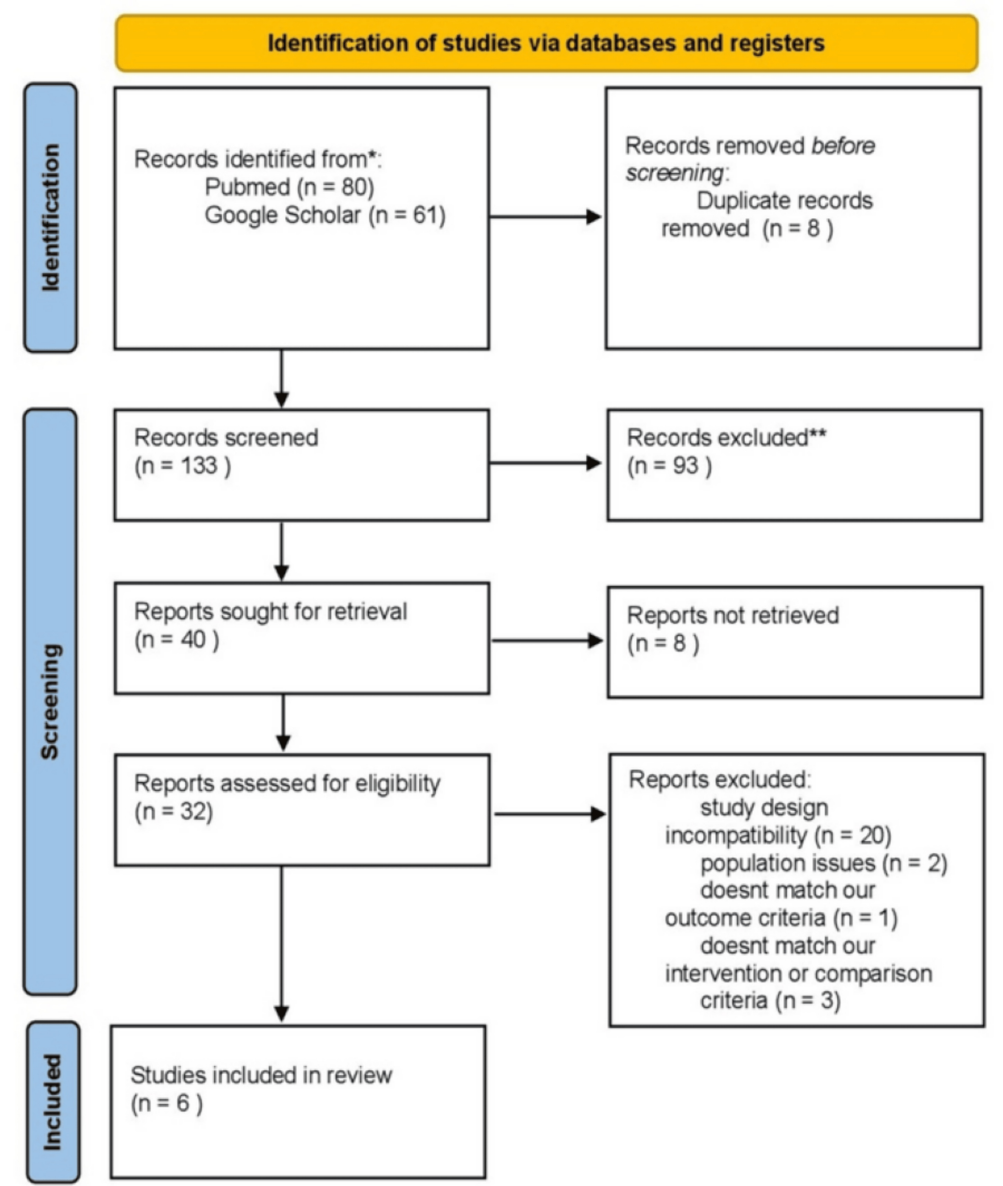
PRISMA flow diagram PRISMA: Preferred Reporting Items for Systematic Reviews and Meta-Analyses *Consider, if feasible to do so, reporting the number of records identified from each database or register searched (rather than the total number across all databases/registers). **If automation tools were used, indicate how many records were excluded by a human and how many were excluded by automation tools.

For inclusion in this narrative review, studies were required to involve patients diagnosed with CRS who had undergone ESS and to evaluate postoperative topical corticosteroid therapy. Only original research articles, specifically randomized controlled trials, prospective or retrospective cohorts, and cross-sectional studies were considered. Reported outcomes of interest included clinical recovery, patient-reported outcomes, revision surgery rates, and safety profiles. Studies were excluded if they did not focus primarily on CRS, involved mixed populations without clear subgroup reporting, or investigated corticosteroid use in non-surgical contexts. In addition, papers that lacked postoperative healing or safety outcomes or were non-original in nature (e.g., case reports, editorials, narrative reviews) were not considered. The process of identification, screening, eligibility assessment, and final inclusion of studies is summarized in a flow diagram (Figure 1).

Process of Screening and Data Extraction

Titles and abstracts were initially screened by one reviewer using the Rayyan web platform for literature reviews [13]. Full-text assessment was subsequently conducted by two independent reviewers. Relevant information was extracted by multiple reviewers and included the following: Study ID (first author, year), article title, journal name, study design (e.g., randomized trial, cohort study), year and country of publication, sample size, group distribution, patient demographics (age, sex, comorbidities, smoking status), follow-up period, intervention characteristics, control group details, duration of treatment, primary and secondary outcomes, tools of measurement, and time points of assessment. Extracted data were cross-checked to prevent duplication and ensure accuracy.

Assessment of Quality and Bias Risk

Risk of bias was appraised using validated tools appropriate for each study design. For RCTs, the Cochrane Collaboration’s Risk of Bias (ROB) tool was applied, assessing six domains: selection bias, performance bias, detection bias, attrition bias, reporting bias, and other potential sources of bias. Each study was rated as having low, high, or unclear risk depending on domain-level evaluation. Two reviewers independently performed the bias assessment, with consensus achieved through discussion when discrepancies arose [14]. Table 1 presents the evidence of the included studies.

**Table 1:** Evidence table of the included studies

Article ID	Author + Reference number	Year	Location	Sample size	Intervention/exposure	Outcome measures	Results (including effect estimates or relevant statistics)
1	Murr et al. [9]	2010	United States	43 patients	The drug-eluting stent is composed of polylactide-co-glycolide. A total dose of 370 ug of MF is blended into the polymer structure of polylactide-co-glycolide, which releases the MF by diffusion in a controlled fashion over approximately 30 days, and the stent is self-expanding	Inflammation (ethmoid sinus, graded on 100-mm visual analog scale VAS). Polyp formation (graded on a 5-point categorical scale). Adhesion formation (graded on a 5-point categorical scale; dense or severe = significant adhesion). Middle turbinate position (graded on a 4-point categorical scale). Device-related adverse events (safety). Systemic safety (plasma mometasone furoate concentrations + cortisol levels).	Inflammation (VAS scores): Day 21: Treatment 23.2 vs Control 35.3 → difference −12.0, p = 0.0032. Day 30: Treatment 20.2 vs Control 30.1 → difference −11.2, p = 0.0011. Day 45: Treatment 15.9 vs Control 24.0 → difference −8.8, p = 0.0022. Day 60: Treatment 12.0 vs Control 17.5 → difference −5.1, p = 0.0855 (not significant) Polyp formation (day 30): Treatment: 18.4% (7/38). Control: 36.8% (14/38) p = 0.0391. Significant adhesions: Treatment: 5.3% (2/38) Control: 21.1% (8/38) p = 0.0313. Middle turbinate lateralization: Treatment: 5.3% (2/38). Control: 15.8% (6/38) p = 0.2188 (not significant). Oral steroid prescription (if polyps by day 30): With polyps: 6/16 received oral steroids. Without polyps: 2/22 received oral steroids. Odds ratio = 6.0 (95% CI 1.02–35.3), p ≈ 0.05. Systemic safety: Plasma mometasone furoate <30 pg/mL at all time points (below quantification limit). Cortisol levels within normal range → no adrenal suppression. Device-related adverse events: None reported.
2	Sood et al. [15]	2023	India	40 patients	Merocel packs were inserted in the bilateral nasal cavities and infiltrated with 4mL triamcinolone (40mg/mL) in one nasal cavity (treatment group) and 4 mL normal saline in the other (control group).	Lund–Kennedy Endoscopic Score (LKES) and Perioperative Sinus Endoscopy (POSE) score at weeks 1, 2, 4, 12; component analysis for crusting, polyp, edema, discharge, scarring	Significant improvement in LKES overall at weeks 1 (p=0.001), 2 (p=0.008), and 12 (p=0.000) but not week 4; component analysis showed significant differences for crusting (week 12), polyp (week 12), edema (week 1), discharge (weeks 1 & 12), no consistent difference in scarring. POSE score significantly better in treatment at weeks 1 (p=0.001), 2 (p=0.029), and 12 (p=0.026) but not week 4. Authors conclude triamcinolone-soaked packs improve early and 3-month postoperative healing compared to saline packs.
3	Huang et al. [16]	2020	China	181 patients	The treatment-side sinus cavity received one bioabsorbable steroid-eluting sinus stent, whereas the contralateral side received the Nasopore pack (Stryker Corp) in a randomized fashion at the end of the ESS.	1 - Need for postoperative surgical interventions. 2 - Polyp formation. 3 - MT lateralization. 4 - Adhesion	1 - The need for postoperative interventions in the ethmoid sinus was significantly lower on the steroid-eluting sinus stent sides at 14.38% and 33.7% compared with the Nasopore sides at 75% and 66.3% (both p < 0.0001). 2 - Polyp formation was significantly lower on the steroid-eluting sinus stent sides at 16.57%, 22.65%, and 6.78% compared with the Nasopore sides at 44.75%, 54.14%, and 25.42% (both p < 0.0001). 3 - Severe adhesion (grades 3-4) was significantly lower on the steroid-eluting sinus stent sides than on the Nasopore sides at postoperative day 90 (7.63% vs 25.42%; p=0.0003). 4 - MT lateralization shows no significant difference between steroid-eluting stent sides and the Nasopore sides.
4	Kothiwala et al. [17]	2021	India	66 patients	High-volume nasal irrigation with budesonide at a dose of 1 mg twice daily for 12 weeks, compared to a control group receiving normal saline irrigation. The intervention was initiated after removal of nasal packing following ESS.	1 - The Sino-Nasal Outcome Test (SNOT-22) score 2- Lund-Kennedy endoscopic scores 3- NCCT PNS score 4-safety parameters including serum cortisol levels and intraocular pressure.	1 - SNOT-22 scores decreased significantly in the budesonide group (53.73 ‡ 15.75 - 21.15 ‡ 13.52) compared to control (52.54 # 16.31 -> 30.06 ‡ 18.16), with 81.8% of budesonide-treated nasal cavities showing improvement versus 72.7% in controls (p < 0.0001). 2 - Lund-Kennedy scores improved significantly in the budesonide group (6.74 # 1.8 - 2.77 = 1.4) compared to control (6.53 ‡ 1.33 - 3.93 ‡ 1.6), with 92.4% of budesonide-treated nasal cavities showing improvement versus 84.8% in controls (p < 0.0001). 3 - Plasma cortisol levels remained within the normal range (5-25 pg/di) and no adrenal suppression was observed; intraocular pressure also remained stable within normal limits (12-22 mmHg) throughout the 12-week intervention.
5	Han et al. [18]	2014	USA	100 patients	Steroid-eluting sinus implant composed of a bioabsorbable polymer and has a self-expanding, non-obstructive design	1 - Redction in bilateral polyp grade 2-nasal obstruction/congestion & ethmoid sinus obstruction	At 3 months, treatment group had greater reduction in bilateral polyp grade (p = 0.016) and ethmoid obstruction (p = 0.001) vs. control. 60% of treatment vs 33% of control had ≥1-grade polyp reduction; 42% vs 9% had ≥2-grade reduction. Nasal obstruction/congestion improved by −1.33 ± 1.5 vs −0.67 ± 1.5 (p = 0.137 overall; p = 0.025 in subgroup with grade ≥2 bilaterally). 53% of treatment vs 23% of control no longer indicated for ESS. No serious adverse events; only 2 mild implant-related events; no ocular complications.
6	Forwith et al. [19]	2016	USA	100 patients	Steroid-eluting sinus implant (1350 μg mometasone furoate, 90-day release); sham procedure (implant inserted then removed); both groups used mometasone furoate nasal spray ( 100 μg/nostril daily)	Endoscopic polyp grade (5-point Meltzer scale); percent ethmoid sinus obstruction (VAS 0–100); nasal obstruction/congestion score (0–5); NOSE score (5 questions, 0–4 scale); device-related adverse events; ocular safety (intraocular pressure, cataracts); need for oral steroids or revision ESS.	1 - The implant was successfully placed in 106/106 sinuses (100%); 2 - Polyp grade improvement was significantly greater with the implant (p < 0.001); 3 - Ethmoid obstruction (VAS) improvement was also significantly greater with the implant (p < 0.001); 4 - Nasal obstruction/congestion score reduction was greater in the implant group (p = 0.007); 5 - NOSE score improvement was greater in the implant group (p = 0.024); 6 - The requirement for oral steroids was lower in the implant group (p = 0.041); 7 - The requirement for revision ESS was also lower in the implant group (p = 0.036); 8 - Safety analysis showed no significant differences in intraocular pressure or cataracts, and device-related adverse events were few and non-serious.

Results

Narrative Synthesis of the Included RCTs

This systematic research yielded a total of 141 papers, with 80 found in PubMed and 61 in Google Scholar (the first 20 pages). After removing duplicates using Rayyan, eight papers were excluded. Four reviewers individually screened the titles and abstracts of 137 papers on Rayyan. Of these, 40 full-text papers were reviewed, 20 articles were excluded due to study design incompatibility, eight articles were excluded due to unavailable full text, two articles were excluded due to population issues, one article was excluded because it does not match our outcome criteria, three articles were excluded because it does not match our intervention or comparison criteria, and only six RCTs published between January 2011 and March 2023 were included in this systematic review. These studies were conducted across three countries: three in the United States, two in India, and one in China.

Description of the Characteristics of the Included Studies

Across all the included studies, a total of 530 patients were studied, with sample sizes ranging from 40 to 181 [15,16]. The age of participants ranged from 15 to 80 years, with the mean age varying from 33 to 49.7 years. The distribution of sex across the studies showed a predominance of male participants, particularly in the study conducted by [16,17]. The outcomes measured included inflammation, polyp formation, adhesions, and middle turbinate (MT) position. Postoperative interventions, MT lateralization, polyp formation, and adhesions were assessed, along with subjective improvements using the Sino-Nasal Outcome Test (SNOT)-22 scores and the Lund-Kennedy classification. Significant improvements were noted in nasal obstruction/congestion, bilateral polyp grade, and ethmoid sinus obstruction. Studies provided a detailed account of the interventions. The interventions included various drug-eluting stents, steroid irrigation, and sinus implants. One stent composed of a bioabsorbable polylactide-co-glycolide polymer, releasing 370 µg of mometasone furoate (MF) over 30 days, controlled drug release, and self-expansion [9]. Another stent, also made of bioabsorbable polymer, was coated with 652 µg of MF [16]. In addition, patients received 1 mg of budesonide via transnasal irrigation twice daily for 12 weeks [17]. A steroid-eluting sinus implant, made of bioabsorbable polymer with a self-expanding, non-obstructive design, was also evaluated [18,19]. Postoperative healing was further compared between Merocel packs infiltrated with 4 mL triamcinolone (40 mg/mL) in one nasal cavity [15]. Follow-up durations ranged from three to six months, with assessments occurring at multiple time points.

Patient-Reported Outcomes, Complications, and Clinical Outcomes

In the 86 sinuses where stents were deployed, the drug-eluting stent demonstrated a significant reduction in inflammation at days 21-45 compared to control (p < 0.003). There was also a statistically significant reduction in the frequency of polyp formation (p = 0.0391) and significant adhesion (p = 0.0313). Although there was a reduced frequency of middle turbinate lateralization, this result did not reach statistical significance. No device-related adverse events were reported [9].

On postoperative day 30, the percentage of patients requiring postoperative interventions was significantly lower in the steroid-eluting stent group compared to the Nasopore group (14.38% vs. 75%; p < 0.0001). A similar trend was seen on day 90 (33.7% vs. 66.3%; p < 0.0001). The frequency of polyp formation was also significantly lower in the steroid-eluting group on postoperative days 14, 30, and 90 (p < 0.0001 for all time points). Severe adhesion (grades 3-4) was significantly reduced by day 90 in the steroid-eluting stent group (7.63% vs. 25.42%; p = 0.0003). No significant difference in MT lateralization was observed between groups [16].

Patients irrigating with budesonide showed a significantly higher rate of improvement in SNOT-22 scores compared to those irrigating with saline (81.8% vs. 72.7%).

Quantitative analysis of SNOT-22 scores showed a significant reduction from 53.73 (15.75) to 21.15 (13.52) in the budesonide group, with a significant difference between the case and control groups (p = 0.0001). Endoscopy scores were also significantly improved in the budesonide group (p = 0.0001) [19].

At six months, treated patients experienced a significant improvement in Nasal Obstruction Symptom Evaluation (NOSE) scores (p = 0.021) and a twofold improvement in nasal obstruction/congestion scores. Endoscopic findings showed a significant reduction in ethmoid sinus obstruction (p < 0.001) and bilateral polyp grade (p = 0.018) compared to controls. A subset of patients with a baseline polyp burden demonstrated a significant reduction in ethmoid sinus obstruction (p = 0.010) and polyp grade (p = 0.049) [19].

At three months, treated patients had significantly lower polyp grades (p = 0.0269) and reduced ethmoid sinus obstruction (p = 0.0001) compared to controls. The mean nasal obstruction/congestion score also improved, but this was not statistically significant (p = 0.1365). In patients with a greater polyp burden (grade 2 bilaterally), there was a statistically significant improvement in congestion scores (p = 0.025). No serious adverse events were reported [18].

There was significant improvement in the Lund-Kennedy score for crusting and polyp at week 12, edema at week one, and nasal discharge at weeks 1 and 12 (p < 0.05). However, no significant improvement was observed in scarring at any postoperative time point. POSE scores improved significantly at weeks one, two, and 12, except at week four [15].

Analyzing Biases, Assessing Quality, and Determining the Level of Evidence

The risk of bias was assessed using the Cochrane Collaboration RoB tool [14]. All included studies overall had a low risk of bias. Common sources of bias included a lack of blinding in some studies, although most trials were well-randomized and controlled. Across all studies, Han et al. [18] reported that clinical investigators were not blinded during the endoscopic grading process, though immediate grading was done to avoid bias. Sood et al. [15] reported limitations because of the short-term follow-up study, and the sample size was small. The overall quality of evidence was determined to be moderate, given that most studies were well-designed RCTs with consistent findings across interventions. The level of evidence suggests that, overall, the use of drug-eluting stents and budesonide irrigation showed significant improvements in outcomes such as polyp formation, sinus obstruction, and postoperative interventions across the studies. The results were consistently significant across various time points, especially in reducing polyp recurrence and adhesion formation. Most studies demonstrated a significant improvement in nasal obstruction scores and sinus endoscopy findings. No device-related adverse events or significant systemic side effects were reported.

Discussion

The purpose of this narrative review was to summarize evidence from RCTs on the role of topical corticosteroids in improving outcomes for patients with CRS undergoing ESS. The included studies consistently demonstrated that topical corticosteroid use was associated with better symptom control, enhanced postoperative healing, and improved quality of life, without major systemic side effects. This strengthens the overall safety profile of localized corticosteroid delivery.

The findings of this review align with earlier reports in the literature. Murr et al. [9] showed that a drug-eluting stent releasing 370 µg of mometasone furoate over 30 days reduced postoperative inflammation, supporting the therapeutic benefits observed across studies. Similarly, Sood et al. [15] found that triamcinolone-soaked nasal dressings significantly improved Lund-Kennedy endoscopy scores (LKES) and POSE scores, with reductions in crusting and edema, while maintaining a favorable safety profile. A further consistent observation across three trials [16,19] was the reduction in polyp recurrence and improvements in bilateral polyp grade following topical corticosteroid use, suggesting that these therapies may also help decrease the likelihood of revision surgery.

While generally well tolerated, minor adverse effects, such as nasal discomfort, unpleasant odor, or episodes of sinusitis, were reported in some studies [16,18,19]. These events were infrequent and did not outweigh the therapeutic benefits. Importantly, none of the reviewed trials described significant systemic complications, further reinforcing the localized safety of these interventions.

Despite the overall strength of evidence, certain limitations should be acknowledged. Differences were noted across trials in the choice of corticosteroid formulation, dose, route of administration, outcome measures, and follow-up durations, which makes direct comparison difficult. Additionally, five studies permitted the use of adjunctive therapies, such as antibiotics, saline irrigation, nasal sprays, or mucolytics, which could have influenced reported outcomes. Some trials also involved relatively small patient cohorts, restricting the generalizability of their findings.

Han et al. [18] reported the possibility of observer bias in endoscopic grading, as investigators were not blinded; although immediate assessments were used to mitigate this limitation, the potential for bias cannot be fully excluded. Future research should therefore aim for larger sample sizes, blinded assessments, and more standardized protocols in terms of intervention type, dosage, and outcome reporting. Such measures would enhance comparability across studies and improve the reliability of conclusions.

A notable limitation across the included RCTs is the heterogeneity in corticosteroid formulations, dosages, routes of administration, outcome measures, and follow-up durations. This variability reduces the comparability between studies and limits the generalizability of the findings to broader clinical practice. Table 2 provides the bias assessment for the included studies.

**Table 2:** Bias assessment for the included studies RCT: randomized controlled trials; RoB: risk of bias

Study ID	Authors/Year	Study Type	Risk of Bias Tool	Selection Bias	Performance Bias	Detection Bias	Attrition Bias	Reporting Bias	Other Bias
Forwith, 2016	Forwith et al., 2016 [19]	RCT	Cochrane RoB	Unclear	Low	Low	Low	Low	None
Han, 2014	Han et al., 2014 [18]	RCT	Cochrane RoB	Low	Low	Low	Low	Low	Clinical investigators performing endoscopic grading were not blinded to the treatment assignment. We attempted to minimize the potential for bias in these assessments by requiring instantaneous grading at each study visit.
Sood, 2023	Sood et al., 2023 [15]	RCT	Cochrane RoB	Unclear	Low	Low	Low	Low	Short-term follow-up study and the sample size was small.
Kothiwala, 2021	Kothiwala et al., 2021 [17]	RCT	Cochrane RoB	Some concern	Low	Low	Low	Low	None
Murr, 2011	Murr et al., 2011 [9]	RCT	Cochrane RoB	Low	Low	Low	Low	Low	None
Huang, 2020	Huang et al., 2020 [16]	RCT	Cochrane RoB	Low	Low	Low	Low	Low	None

## Conclusions

We carried out a narrative synthesis of RCTs evaluating the use of topical corticosteroids for improving postoperative outcomes after endoscopic sinus surgery in patients with CRS. The findings demonstrated significant improvements in mucosal healing, reduction of inflammation, and lower rates of adhesion formation, with minimal systemic side effects. However, significant heterogeneity exists regarding corticosteroid formulations, dosages, routes of administration, measured outcomes, and follow-up durations. We suggest that further well-controlled trials with standardized treatment protocols are needed to validate these benefits and optimize best practices for postoperative CRS management. Future well-controlled trials with standardized treatment protocols are needed to confirm these benefits and establish best practices for the postoperative management of CRS.
